# Open-aqueduct LOVA, LIAS, iNPH: a comparative clinical-radiological study exploring the “grey zone” between different forms of chronic adulthood hydrocephalus

**DOI:** 10.1007/s00701-022-05215-9

**Published:** 2022-04-27

**Authors:** Giorgio Palandri, Alessandro Carretta, Emanuele La Corte, Giulia Giannini, Matteo Martinoni, Paolo Mantovani, Luca Albini-Riccioli, Caterina Tonon, Diego Mazzatenta, Benjamin D. Elder, Alfredo Conti

**Affiliations:** 1 Department of Neurosurgery, IRCCS Istituto Delle Scienze Neurologiche Di Bologna, Via Altura 3, 40139 Bologna, Italy; 2 Department of Biomedical and NeuroMotor Sciences (DIBINEM), University of Bologna, Bologna, Italy; 3 Neurology Unit (NEUROMET), IRCCS Istituto Delle Scienze Neurologiche Di Bologna, Bologna, Italy; 4 Neuroradiology Unit, IRCCS Istituto Delle Scienze Neurologiche Di Bologna, Bologna, Italy; 5 Functional and Molecular Neuroimaging Unit, IRCCS Istituto Delle Scienze Neurologiche Di Bologna, Bologna, Italy; 6 Programma Neurochirurgia Ipofisi - Pituitary Unit, IRCCS Istituto Delle Scienze Neurologiche Di Bologna, Bologna, Italy; 7 Department of Neurologic Surgery, Mayo Clinic, Rochester, MN USA

**Keywords:** Longstanding overt ventriculomegaly in adults (LOVA), Late-onset idiopathic aqueductal stenosis (LIAS), Idiopathic normal pressure hydrocephalus (iNPH), Differential diagnosis, Score

## Abstract

**Purpose:**

The definition of chronic adult hydrocephalus encompasses different pathological entities with overlapping characteristics, including long-standing overt ventriculomegaly in adults (LOVA), late-onset idiopathic aqueductal stenosis (LIAS) and idiopathic normal pressure hydrocephalus (iNPH). The aim of our study was to identify preoperative clinical and radiological features peculiar of these diseases providing some pathophysiology inferences on these forms of hydrocephalus.

**Methods:**

Clinical and radiological preoperative records, type of surgical treatment and clinical outcome of patients with chronic adult hydrocephalus who were surgically treated between 2013 and 2019 were retrospectively reviewed. Univariate and multivariate analyses were performed to evaluate the contribution of each variable to the differential diagnosis.

**Results:**

In total, 105 patients were included: 18 with LOVA, 23 with LIAS and 64 with iNPH. On multivariate analysis, an enlarged cisterna magna and a more severe ventriculomegaly were associated with the diagnosis of LOVA, while an older age and DESH with iNPH. LIAS patients tend to have an higher prevalence of raised ICP symptoms. Based on that, a clinical and radiological scoring system was developed to distinguish between iNPH and no iNPH cases. A precise cut-off value with a sensitivity of 95.1% and a specificity of 90.6% was identified.

**Conclusions:**

LOVA, LIAS and iNPH are different forms of chronic adulthood hydrocephalus and present different and peculiar clinical and radiological features, with an impact on the treatment and outcome prediction. The implementation of a clinical-radiological score for differential diagnosis may help the differentiation. Further studies are warranted.

**Supplementary Information:**

The online version contains supplementary material available at 10.1007/s00701-022-05215-9.

## Introduction

The definition of chronic adult hydrocephalus encompasses different pathological entities with overlapping characteristics. Long-standing overt ventriculomegaly in adults has been firstly described as a distinct pathological entity by Oi et al. in 1996 [34]. There are, however, other heterogeneous conditions described as “arrested hydrocephalus”, “occult hydrocephalus” or “compensated hydrocephalus” without consensus about specific features of these pathological entities. Thus, diagnostic criteria are a matter of debate even among expert clinicians [10, 17, 28, 29, 39, 40].

For instance, aqueductal stenosis has been considered as a common feature in LOVA patients, but its pathogenetic role is matter of debate [37, 42]. Thus, some authors hypothesized two subtypes of LOVA according to the patency of the aqueduct, with different aetiology [18]. Indeed, the aqueductal stenosis seems to characterize a specific subtype of adulthood hydrocephalus that can be defined as late-onset idiopathic aqueductal stenosis (LIAS) [10, 29, 44].

It is thus not clear if LIAS should be considered a subgroup of LOVA patients and if LOVA with aqueductal stenosis have different clinical, diagnostic and treatment features when compared to those without.

Furthermore, idiopathic normal pressure hydrocephalus (iNPH), a better recognized and separate clinical entity, also shares many radiological and clinical features with LOVA, especially with an open aqueduct [5, 6, 14].

The differential diagnosis between these three diseases (LOVA, LIAS, iNPH) is not trivial, as different forms of hydrocephalus may benefit from different surgical procedures, as endoscopic third ventriculostomy (ETV) or ventriculo-peritoneal shunting (VPS) [2, 7, 12, 18, 19, 21, 29, 36, 38, 41, 42]. Accordingly, a more accurate classification of chronic adulthood hydrocephalus is desirable to improve our ability to plan surgical treatment and predict patient outcome [8].

The aim of the present study was to identify specific preoperative clinical and neuroradiological features in a retrospective comparative cohort of patients with adult hydrocephalus, in order to define the major clinical and radiological characteristics of LOVA, LIAS and iNPH patients, outlining also their clinical outcome after treatment. Moreover, according to literature, the major discrimination in surgical treatment of the aforementioned pathologies is between LOVA and LIAS, for which ETV as a first choice treatment is suggested, and iNPH, which requires VPS or other forms of CSF shunting. We also explored the feasibility of a diagnostic score to support the differential diagnosis between iNPH and obstructive hydrocephalus patients.

## Methods

### Study design, setting and inclusion criteria

We retrospectively selected patients consecutively referred to the Department of Neurosurgery of the IRCCS Istituto delle Scienze Neurologiche di Bologna (ISNB) between July 2013 and December 2019 (78 months) and who underwent ETV and/or VPS for adult hydrocephalus.

All surgical procedures were performed by one single surgeon leading the hydrocephalus management group (G.P.). Included patients were categorized in LOVA, LIAS and iNPH subcohorts, respectively.

The following criteria were further required for being included in this study: (1) age > 18 years old; (2) exclusion of secondary hydrocephalus, such as post-traumatic, post-infectious, post-hemorrhagic or caused by neoplasms affecting the patency of CSF pathways; (3) absence of foramina of Monro, Luschka and Magendie stenosis causing hydrocephalus; (4) no previous neurosurgical procedures performed; (5) available complete preoperative imaging and clinical history.

### Cohort assignment

#### LOVA

Characteristics of this cohort have been extensively described in a previous paper [36]. In this present study, we included in the group an open-aqueduct LOVA patients’ cohort consecutively treated at our institution. All the patients had MRI evidence of CSF flow through the aqueduct and their clinical and radiological parameters were consistent with LOVA diagnosis according to Ved’s criteria (Table 1) [42].

**Table 1:** Clinical-radiological criteria for the diagnosis of LOVA according to Ved et al. [42]

1.Clinical symptoms of hydrocephalus developing in adulthood—e.g. headaches, cognitive decline, imbalance, gait disturbance, psychological disturbance, visual deterioration/diplopia;
2.Macrocephaly defined by head circumference > 98th percentile in adulthood (male 53.8 cm; female 52.9 cm);
3.Overt tri-ventriculomegaly (lateral and third ventricles) on neuroimaging, with cortical sulcal effacement and/or destruction of the sella turcica as evidence of long-standing ventriculomegaly;
4.Absence of a secondary cause for aqueductal stenosis in adulthood (e.g. previous meningitis, subarachnoid haemorrhage)

#### LIAS

In the second group, we allocated patients without CSF flow through aqueduct in phase-contrast (PC) MRI sequences, no turbulence void signal in T2-weighted images, and evidence of complete aqueductal stenosis in midsagittal thin-slice sequences, as established in literature [10, 29]. These patients were diagnosed as affected by LIAS.

#### iNPH

A third group included iNPH patients whose data were retrospectively analysed from an ongoing prospective observational study (the Bologna PRO-HYDRO study) database. Characteristics of this cohort have been also extensively reported in previous papers [11, 30]. Briefly, all the patients with clinical suspicion of chronic primary adulthood hydrocephalus were extensively investigated with a specific clinical and diagnostic assessment, including 3 T brain magnetic resonance imaging (MRI). iNPH clinical assessment was conducted on 5 consecutive days in our inpatient clinic and included standardized clinical evaluations, neuropsychological assessments, tap test and instrumental gait analysis before and after (24 h and 72 h) the tap test. On the basis of these findings, a multidisciplinary team (neurologists, neurosurgeons, neuropsychologists, neuroradiologists, physiatrists, engineers in movement science and nurses) established eligible patients for VPS placement. All of them were consistent with the diagnosis of “probable iNPH” according to the 2005 guidelines [38].

### Variables

The following preoperative clinical and radiological features were collected from clinical and radiological records: (1) age and sex; (2) symptomatology on admission as headache, nausea, papilledema, gait disturbance, urinary incontinence, cognitive impairment; (3) cranial circumference (measured in centimetres); (4) neuroradiological features as evidence of sellar bone distortion, empty sella (defined as at least 66% of pituitary height loss, grades IV–V according to Yuh WTC et al. [46]), DESH sign [32], bulging of the third ventricle floor [16, 33], an enlarged cisterna magna measuring ≥ 10 mm on mid-sagittal images [4], Evans’ index, third ventricle width (measured in millimetres), callosal angle and tentorial angle. Moreover, the type of surgical procedures the patients underwent and their clinical status at the last follow-up visit were also collected. The outcome was classified as “improved” if any improvement in at least one symptomalogical domain was reported (with stability of the remaining ones), “stable” if complete stability or halting of the symptomatological progression were described and “worsened” if the patient suffered deterioration in at least one domain of the preoperative status.

The qualitative radiological parameters were independently evaluated by three blinded researchers (A.C., E.L.C. and M.M.) and discussion was performed in case of disagreement. For continuous data collected, the mean among the three measurements was calculated and included. Data were reported according to the STROBE guidelines for observational studies.

### Diagnostic score

The proposed diagnostic score is shown in Table 2. Every patient included in our study, according to his clinical and radiological features, received a grade for each domain, which are added up to obtain a single score ranging from 0 to 17. In contrast to the “iNPH” cohort, in the “no iNPH” cohort were included both LIAS and LOVA patients.

**Table 2:** Diagnostic score proposed in our study to differentiate patients with iNPH, potentially benefiting from a VPS, from patients with obstructive hydrocephalus (LOVA and LIAS) requiring ETV. The grades assigned for every feature are added up to obtain a single score

Variable	Cut-off	Score
Age, y	≤ 72	2
	> 73	0
Cranial circumference, cm	≤ 56	0
	> 56	1
Evans’ index	≤ 0.4	0
	> 0.4	1
Third ventricle width, mm	≤ 18	0
	> 18	1
Sellar bone distortion	Yes	2
	No	0
Third ventricle floor bulging	Yes	2
	No	0
DESH	Yes	0
	No	2
Headache	Yes	2
	No	0
Nausea and vomit	Yes	1
	No	0
Gait disturbances	Yes	0
	No	1
Urinary incontinence	Yes	0
	No	1
Cognitive impairment	Yes	0
	No	1

### Data sources

Clinical and neuroradiological data were retrospectively collected from our institution digital records and included in an-hoc anonymized database.

### Ethics approval

The aforementioned prospective PRO-HYDRO study was approved by the Ethics Review Board of our Institute (Cod. CE: 14,131, 23/02/2015.) and all patients consented to the use of their clinical records for research. On the other hand, ethics committee approval was waived for the retrospective observational part of this study.

### Statistical methods

The statistical analysis was performed with IBM SPSS Statistics Version 28.0.1.0 (IBM Corp. Released 2021. IBM SPSS Statistics for Mac. Armonk, NY: IBM Corp.). Normal distribution was analysed with the Shapiro–Wilk test. According to their normal or not normal distribution, continuous variables (age, cranial circumference, tentorial angle, callosal angle, Evans’ index and third ventricle width) were compared using parametric one-way ANOVA and non-parametric Kruskal–Wallis *H* test, respectively. Bonferroni and Mann–Whitney *U* post hoc test was performed to evaluate intergroup variability. Categorical variables (sex, sellar bone distortion, empty sella, bulging of third ventricle floor, enlarged cisterna magna and all the clinical manifestations) were catalogued in contingency tables according to the patients’ cohort and compared with Freeman-Halton extension of the Fisher exact probability test. LOVA and LIAS outcome comparison was also compared with two-tailed Fisher exact probability test. Significant parameters on univariate analysis were compared with multivariate logistic regression. Furthermore, cutoff values between different pathological entities were determined using different receiver operating characteristic (ROC) curves, and accuracy rates were calculated measuring areas under the ROC curves (AUC). AUCs < 0.5 were excluded. The Youden Index (*J*) was used to determine the sensitivity and specificity rates of the cutoff values between populations. Odds ratios (OR) and confidence intervals (CI) of different cutoff values were determined using contingency tables.

Similarly, cutoff values of the continuous variables included in the diagnostic score were also determined using ROC curves and AUCs. Variables with AUC < 0.5 were excluded from the diagnostic score. The cutoff score between “iNPH” and “no iNPH” population, its sensitivity and specificity was also calculated with Youden Index. Odds ratios (OR) and confidence intervals (CI) of different cutoff values were determined using contingency tables.

The *p*-value was assumed to be statistically significant when ≤ 0.05.

## Results

### Demographics

A total of 192 patients in the selected timespan were considered and analysed for the purpose of this study. Twenty-two (11.5%) lacked complete clinical and/or radiological data and were therefore excluded, as well as 58 (30.2%) who were treated for secondary hydrocephalus and 7 (3.6%) who had undergone previous neurosurgical procedures. Of 105 patients included in this study, 18 (12 males and 6 females) were assigned to the LOVA cohort, 23 (12 males and 11 females) to the LIAS cohort and 64 (33 males and 31 females) to iNPH group.

Median age at the time of surgery significantly differed among the three groups: 70 years (64–72) for LOVA, 59 (38–68.5) years for LIAS and 75.5 (73–78.75) for iNPH, *p* < 0.001 (Table 3).The univariate analysis demonstrated that LOVA were significantly older than LIAS (*p* = 0.03) and significantly younger than iNPH patients (*p* < 0.001). ROC analysis reported a cutoff of 59.5 years to discriminate LOVA and LIAS, *J* = 0.41, OR 1.974 (Supplementary Table 1 and Table 4). No difference in gender rates was identified.

**Table 3:** Clinical features of the three cohorts. Data are expressed as *n* (%) or median (interquartile range)

Variable	LOVA	LIAS	iNPH	*p*-value
	*n* = 18	*n* = 23	*n* = 64	
Age, y	70 (64–72)	59 (38–68.5)	75.5 (73–78.75)	< 0.001^a^
Males, *n*	12 (66.7)	12 (52.2)	33 (51.6)	0.16
Cranial circumference, cm	58.5 (57–59.75)	57 (55.5–58.5)	56 (55–58)	0.001^a^
Headache, *n*	3 (16.7)	7 (30.4)	1 (1.6)	< 0.001^a^
Nausea and vomit, *n*	2 (11.1)	2 (8.7)	0 (0)	0.02^a^
Gait disturbances, *n*	17 (94.4)	17 (73.9)	64 (100)	< 0.001^a^
Urinary incontinence, *n*	15 (83.3)	10 (43.5)	57 (89.1)	< 0.001^a^
Cognitive impairment, *n*	12 (66.7)	9 (39.1)	55 (87.5)	< 0.001^a^

^a^Statistically significant *p*-value on univariate analysis

**Table 4:** Comparison of the single cohorts with univariate and multivariate analysis according to the different radiological and clinical parameters. Only significant *p*-values and corresponding odds ratios (OR) with 95% confidence intervals at multivariate analysis have been reported

	LOVA vs. LIAS			LOVA vs.iNPH			LIAS vs.iNPH		
Variable	Univariate	Multivariate		Univariate	Multivariate		Univariate	Multivariate	
	*p*-value	*p*-value	OR (95% CI)	*p*-value	*p*-value	OR (95% CI)	*p*-value	*p*-value	OR (95% CI)
Age, y	0.03	-	-	< 0.001	-	-	< 0.001	-	-
Cranial circumference, cm	-	-	-	0.001	-	-	-	-	-
Evans’ index	0.02	-	-	< 0.001	-	-	-	-	-
Third ventricle width, mm	0.01	0.007	5.90 (1.46–23.8)	0.002	0.001	22.13 (1.01–482.85)	-	-	-
Callosal angle, °	0.04	-	-	-	-	-	-	-	-
Sellar bone distortion, *n*	-	-	-	0.002	-	-	0.02	-	-
Third ventricle floor bulging, *n*	-	-	-	0.005	-	-	< 0.001	-	-
Enlarged cisterna magna, *n*	< 0.001	< 0.001	1.67 × 10^11^ (7.64 × 10^9^ − 3.64 × 10^12^)	< 0.001	0.002	6.41 (1.75–23.49)	0.002	-	-
DESH, *n*	-	-	-	< 0.001	< 0.001	42.12 (5.95–298.05)	< 0.001	< 0.001	236.79 (13.21–4244.05)
Headache, *n*	-	-	-	0.04	-	-	< 0.001	-	-
Nausea and vomit, *n*	-	-	-	0.05	-	-	-	-	-
Gait disturbances, *n*	-	-	-	-	-	-	< 0.001	-	-
Urinary incontinence, *n*	0.03	-	-	-	-	-	< 0.001	-	-
Cognitive impairment, *n*	-	-	-	-	-	-	< 0.001	-	-

### Clinical features

**Fig. 1:**
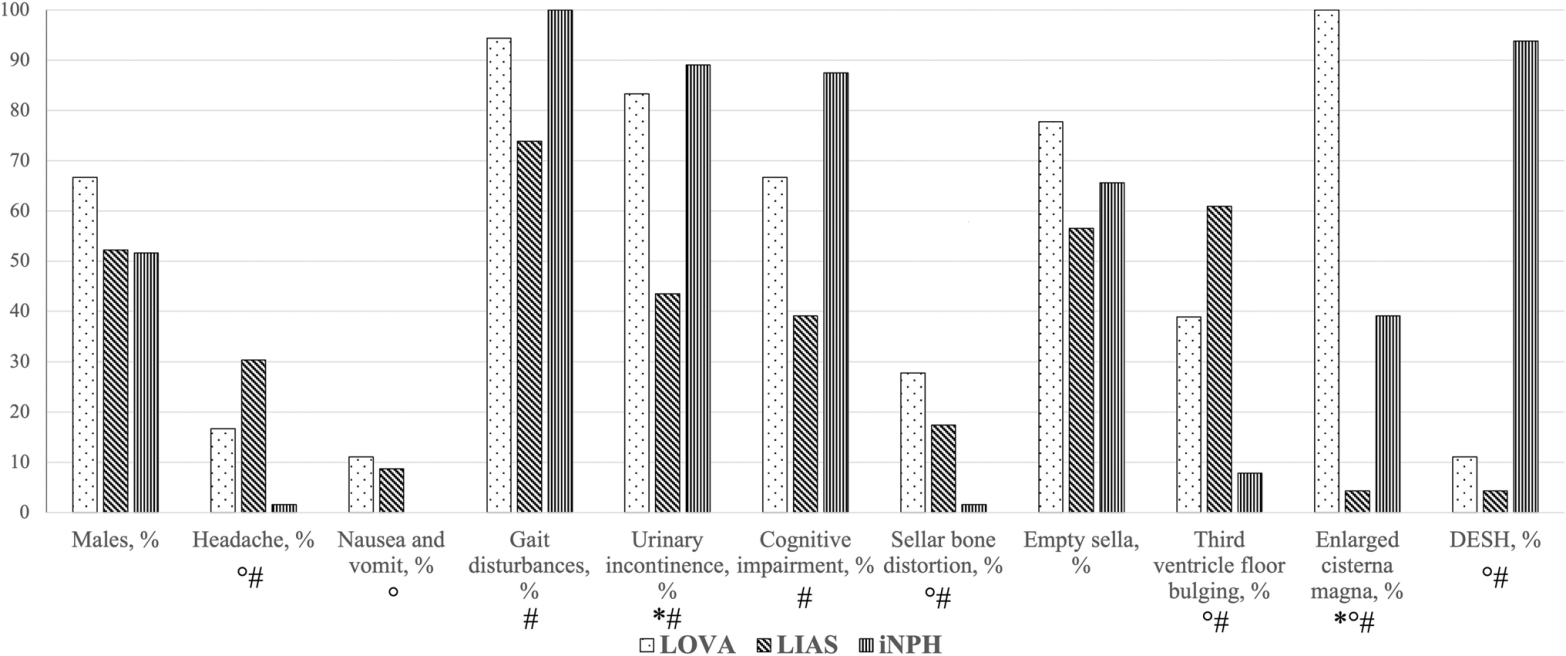
Incidences of categorical neuroradiological and clinical features in the three cohorts are reported. Significant differences according to univariate analysis are also underlined. *LOVA vs. LIAS. °LOVA vs iNPH. #LIAS vs iNPH

Clinical characteristics are summarized and compared in Fig. 1 and Tables 3 and 4. Signs and symptoms of raised ICP were reported in LOVA (16.7% for headache and 11.1% for nausea) and LIAS (30.4% and 8.7%, respectively), while they were absent in iNPH patients. A significant lower occurrence of headache was reported in iNPH group when compared to LOVA and LIAS (*p* = 0.04 and *p* < 0.001, respectively) by univariate analysis. Similar results were found for nausea, with borderline significance (*p* = 0.05) between LOVA and iNPH. Multivariate analysis did not yield significant results.

Incidental papilledema, in our cohort, was found only in one patient suffering from LIAS and therefore was not analysed among the other clinical manifestations.

At least one of the Hakim’s triad symptoms was reported in every iNPH patient, with a constant presence of gait abnormalities and a high prevalence of urinary incontinence and cognitive impairment (89.1% and 87.5% respectively). Symptoms of Hakim’s triad were reported in a slightly smaller proportion of the LOVA cohort, with gait abnormalities present in 94.4% of the patients, urinary incontinence in 83.3% and cognitive impairment in 66.7%. These symptoms were, however, less frequent in LIAS patients, with a prevalence of 73.9%, 43.5% and 39.1% respectively. Univariate analysis showed strong significance comparing Hakim’s triad between LIAS and iNPH cohorts (*p* < 0.001) and when comparing the prevalence of urinary incontinence in LOVA and LIAS (*p* = 0.03).

Median cranial circumference was 58.5, 57 and 56 cm in LOVA, LIAS and iNPH with a statistically significant difference between LOVA and iNPH (*p* = 0.001).

ROC analysis reported a cranial circumference cutoff of 56.5 cm to discriminate between LOVA and iNPH, *J* = 0.36, OR 1.320 (Supplementary Table 1 and Table 4).

### Radiological features

Neuroradiological features of the 3 groups are shown and compared in Fig. 1, Tables 4 and 5, Supplementary Table 1. No significant difference in tentorial angle between cohorts was found. On the other hand, a significant difference in Evans’ index was disclosed among groups (*p* < 0.001). This significance was ascribable to the difference between LOVA and both LIAS (*p* = 0.02) and iNPH (*p* < 0.001). Moreover, ROC analysis determined Evans’ index cutoff values of 0.365 (*J* = 0.37, OR 1.974) and 0.425 (*J* = 0.59, OR 2.456) between LOVA and the other two entities, respectively.

**Table 5:** Radiological features of the three cohorts. Data are expressed as *n* (%) or median (interquartile range)

Variable	LOVA	LIAS	iNPH	*p*-value
	*n* = 18	*n* = 23	*n* = 64	
Tentorial angle, °	50 (48–56.5)	48 (43–50)	48.5 (42.25–56)	0.72
Evans’ index	0.44 (0.4–0.48)	0.38 (0.35–0.41)	0.37 (0.34–0.4)	< 0.001^a^
Third ventricle width, mm	18.5 (14.3–22.8)	12 (11–16)	14 (12–16)	0.001^a^
Callosal angle, °	63 (51–80)	87 (63–100)	69 (59.25–76.75)	0.02^a^
Sellar bone distortion, *n*	5 (27.8)	4 (17.4)	1 (1.6)	0.01^a^
Empty sella, *n*	14 (77.8)	13 (56.5)	42 (65.6)	0.33
Third ventricle floor bulging, *n*	7 (38.9)	14 (60.9)	5 (7.8)	< 0.001^a^
Enlarged cisterna magna, *n*	18 (100)	1 (4.3)	25 (39.1)	< 0.001^a^
DESH, *n*	2 (11.1)	1 (4.3)	60 (93.8)	< 0.001^a^

^a^Statistically significant *p*-value at univariate analysis

We found a significant larger third ventricle width comparing LOVA with LIAS (*p* = 0.01) and iNPH (*p* = 0.002), mirroring the Evans’ index. Furthermore, ROC curves showed a cutoff value of 13.5 mm (*J* = 0.5, OR 2.253) for LOVA vs. LIAS and 18.5 mm (*J* = 0.45, OR 2.429) for LOVA vs. iNPH.

In addition, comparable but not equal results have been reported analyzing the callosal angle. A significant variation between the three groups was found (*p* = 0.02); the post hoc analysis reported a significantly smaller callosal angle (*p* = 0.04) in LOVA compared to LIAS. The iNPH group showed a smaller callosal angle than the LIAS one, without reaching a statistical significance (*p* = 0.06).

Multivariate analysis confirmed the role of ventricular size (i.e. third ventricle width) as an independent predictor of the diagnosis of LOVA.

The prevalence of an empty sella was not found to be significantly different in the three cohorts. On the other hand, sellar bone abnormalities and the bulging of third ventricle floor were reported to be significant features of LOVA and LIAS but not of iNPH. The prevalence of an empty sella was 27.8% in LOVA and 17.4% in LIAS, while it was found only in one iNPH patient. Univariate analysis confirms these findings (*p* = 0.002 and *p* = 0.02, respectively). Similar results were reported for the bulging of the third ventricle floor, as it was found in 38.9% of the LOVA cohort, 60.9% of the LIAS cohort and only 7.8% of the iNPH cohort. Statistical significance on univariate analysis (*p* = 0.005 and *p* < 0.001, respectively) was shown.

A remarkable finding was an enlarged cisterna magna in all the patients of the LOVA cohort, especially when compared to its prevalence in LIAS (4.3%) and iNPH (39.1%). Univariate analysis confirmed the differences between the cohorts with strong statistical significance (*p* < 0.001).

DESH sign, as a well-known typical feature of iNPH, was found in 93.8% of this cohort, with a significantly lower (*p* < 0.001) prevalence in LOVA (11.1%) and LIAS (4.3%).

Further multivariate analysis did not confirm the significance of sellar distortion and bulging of the third ventricle floor, but strongly underlined enlarged cisterna magna (*p* < 0.001 or *p* = 0.002) as a differentiation factor between the three pathologies and DESH (*p* < 0.001) as a typical feature of iNPH.

### Diagnostic score

According to cohort assignment, 64 patients were included in the iNPH cohort and 41 patients in the “no iNPH” cohort. ROC curves identified different cutoffs of the continuous variables, except for callosal angle which showed an AUC of 0.44 and was therefore excluded (Supplementary Table 2). Similarly, contingency tables analysed ORs and CIs of the features included in the proposed diagnostic score (Supplementary Table 3).

**Fig. 2:**
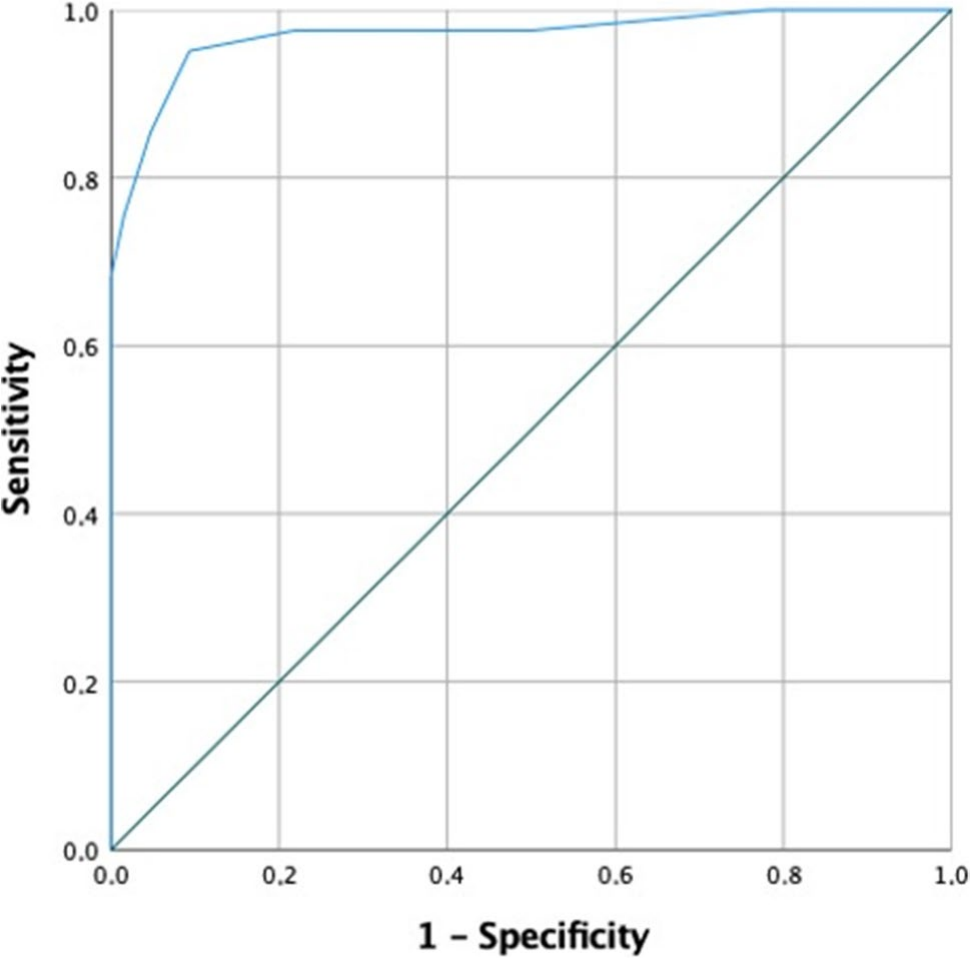
ROC curve describing diagnostic accuracy of the proposed score to differentiate between iNPH and no iNPH patients, AUC = 0.97, standard error = 0.017. Accuracy 94%. Best cutoff value: 3.5, *J* = 0.857, OR 188.5, 95% CI 36.166–982.469. Sensitivity: 95.1%. Specificity: 90.6%

Furthermore, ROC curve analysis, as outlined in Fig. 2, reported a cutoff value of 3.5 to best discriminate between iNPH and no iNPH cohorts (*J* = 0.857, OR 188.5, 95% CI 36.166–982.469). Patients with a diagnostic score of 3 or less belong, with a sensitivity of 95.1% and a specificity of 90.6%, to the iNPH cohort. Conversely, patients with a diagnostic score of 4 or more belong with the same accuracy to the “no iNPH” cohort.

### Clinical outcome

Among LOVA patients, 12 underwent ETV and 6 underwent VPS. Five patients (27.8%) were lost to follow-up, 3 of them belonging to ETV group and 2 to VPS. A clinical improvement at follow-up visit was reported in 6 patients receiving ETV (66.7%), stability in 1 patient (11.1%) and clinical worsening in 2 patients (22.2%). Median follow-up was 38 months (interquartile range 15–67) [36].

In the LIAS cohort, all the patients underwent ETV, with 2 (8.7%) lost to follow-up. A clinical improvement was reported in all patients at a median follow-up duration of 28 months (interquartile range 6–44).

All the patients with a diagnosis of iNPH underwent VPS. Two (3.1%) were lost to follow-up. Clinical improvement was observed in 45 cases (72.6%), stability in 6 cases (9.7%) and worsening in 11 cases (17.7%). Median follow-up was 16 months (interquartile range 6–24).

## Discussion

In this study, a clinical and radiological categorization of patients with chronic hydrocephalus was attempted. Some of those characteristics turned out to have a significant diagnostic value. Among the most relevant, ventriculomegaly, measured by an enlarged third ventricle width and Evans index > 0.40, a cranial circumference > 56.5 cm and an over-represented cisterna magna were distinctive features of LOVA. Moreover, we found a remarkable correlation of an enlarged cisterna magna and upward compressed cerebellar folia with the diagnosis of LOVA. This finding is indeed of value when speculating on the possible physiopathology of this entity and helps understanding why an apparent form of communicating hydrocephalus responds satisfactorily to ETV. We also propose a scoring system to support the differential diagnosis between iNPH and no iNPH based on clinic-radiological characteristics retrieved in our patients. The present comparative study actually completes our previous work whose aim was to shedding some light on the misty differential diagnosis of adulthood chronic hydrocephalus [36].

### The cisterna magna enlargement

**Fig. 3:**
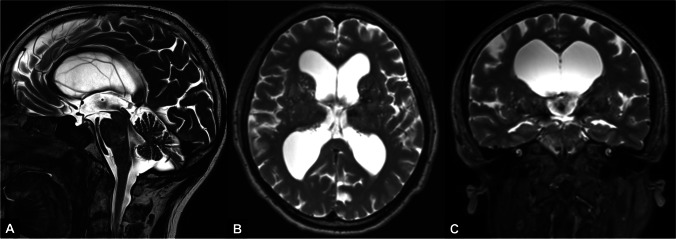
T2-weighted MRI showing landmark features of LOVA in midsagittal (**A**), axial (**B**) and coronal (**C**) view. **A** Empty sella, patent aqueduct with turbulent flow and an enlarged cisterna magna are reported. **B**, **C** Concomitant severe supratentorial ventriculomegaly is observed. Evans Index: 0.47. Third ventricle width: 26 mm

**Fig. 4:**
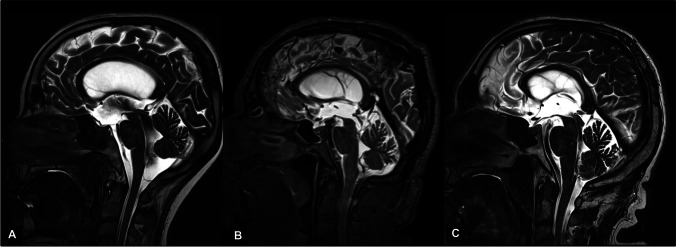
T2-weighted MRI in midsagittal view of the three different types of adult hydrocephalus analysed in our study is compared. **A** LOVA, distorted and empty sella, bulging of third ventricle floor, turbulent flow through the aqueduct and an enlarged cisterna magna with upwards shifted cerebellar vermis are showed. **B** LIAS, CSF flow throughout the aqueduct, which is totally obstructed by a thin septum, is absent. **C** iNPH, with a patent aqueduct, and no abnormalities in the anatomy of sella turcica, third ventricle floor and cisterna magna

We consider the regular evidence of an enlarged cisterna magna in all the patients included in our LOVA cohort as the most relevant finding of our study. This feature is rare in LIAS patients (4.3%) and has a limited prevalence in iNPH patients (38.1%). Furthermore, the continuous turbulent flow starting from the third ventricle, throughout the aqueduct and fourth ventricle until the cisterna magna and the distorted anatomy of the posterior fossa, yet previously described [36], characterized by an upwards shifted cerebellum and compressed caudal folia (Fig. 3), are lacking in the other patients’ cohorts, especially in iNPH when a slightly enlarged cisterna magna could be observed (Fig. 4). According to these results, an enlarged cisterna magna be taken into consideration as a possible diagnostic criteria of open-aqueduct LOVA. This feature is also associated, due to its strongly correlation with LOVA, with a more severe ventriculomegaly as further discussed.

Thus, to our knowledge, this is one of the first studies to correlate supratentorial adult hydrocephalus and the increased volumetry of the cisterna magna as a specific characteristic of the open-aqueduct LOVA subgroup of patients. Our study corroborates the theory of the “intracisternal” obstruction in communicant hydrocephalus with a bulging third ventricle floor, hypothesized by Kehler in 2003 [23] and discussed by Kageyama [22] in 2016 and Al-Hakim in 2019 [1], as peculiar of LOVA, in view of the lack of posterior fossa abnormalities in the other cohorts. Venous stenosis, as already theorized by other authors, could have a possible role in distal CSF blockade and impaired reabsorption, but this does not explain the satisfactory outcomes achieved by ETV in patients without aqueductal stenosis [36, 42].

### The ventricle size

According to our observations, LOVA cases had a cranial circumference significantly greater than iNPH but similar to LIAS. They had a median age of 70, the presence of an enlarged cisterna magna and a symptomatology encompassing raised ICP and Hakim’s triad (Fig. 2).

Moreover, Ibanez-Botella et al. underlined the “overt” ventriculomegaly as a feature of LOVA patients, proposing a minimum Evans’ index of 0.4 as a criterion for their diagnosis [18]. Our study confirms this severe degree of ventriculomegaly in LOVA, especially when compared to the other forms of chronic adulthood hydrocephalus. The two parameters assessing ventriculomegaly, namely the Evans’ index and third ventricle width, have the potential to provide robust differentiation among LOVA and the other two groups. Reasonably, this phenomenon reflects the “long-standing” feature of this pathology, with a slow and almost asymptomatic course for the most part of the patient’s lifespan until late decompensation, and the induced adaptive changes of the brain and the ventricular anatomy [35, 45]. Nonetheless, in our opinion, a cut-off value of 0.4 in the Evans’ index could be too strict for the diagnosis of LOVA and therefore carrying a concrete risk of misdiagnosing some patients, as underlined in our previous analysis [36]. The aforementioned cut-off value, for example, would have excluded one quarter of the analysed LOVA cohort.

### Other parameters

On the other hand, LIAS patients represented the youngest cohort, with a median age at surgery of 59, whereas LOVA patients had a median age of 70 and iNPH a median age of 75.5 (Table 3).

LIAS and LOVA presented higher rates of sellar bone abnormalities and bulging of the third ventricle, although only at univariate analysis, when compared to iNPH. These results are congruous with the obstructive pathogenetic hypothesis of both subtypes of chronic hydrocephalus. iNPH, according to its “normal pressure” definition, lacks these features [32]. The callosal angle, which is a widely accepted parameter to differentiate iNPH from atrophic hydrocephalus and a predictor of outcome [20, 43], was similar between LOVA and iNPH (median values 63 and 69 degrees, respectively), but much higher in LIAS (median 87 degrees). On the other hand and in line with literature, the prevalence of DESH, a recognized and peculiar feature of iNPH, was a characteristic of the iNPH where it was present in 93.8% versus 11.1% in LOVA and 4.3% in LIAS (Table 5 and Fig. 3).

Clinical symptoms caused by raised ICP, like headache and nausea, were present in our LOVA and LIAS cohort, although infrequently (overall less than 30% of prevalence), but substantially absent in iNPH. These results mirror the prevalence of sellar bone changes and third ventricle floor bulging, indeed reflecting obstructive pathogenesis with long-standing raised ICP. Green et al. recently investigated ICP measurements and amplitude in a direct comparison between iNPH and LIAS patients, with the expected finding of a mean higher ICP in the latter cohort, although not critical and lower than the 15 mmHg cut-off, strengthening the hypothesis of a chronically compensated supratentorial CSF accumulation due to aqueductal stenosis [13]. Other authors also underlined the role of ICP pulsatility in the etiopathogenesis of LIAS [9].

As expected, a complete Hakim’s triad was fundamental for the diagnosis of iNPH, with gait disturbance as a constant symptom of that cohort, urinary incontinence found in 89.1% of patients and cognitive impairment in 87.5%.

A similar occurrence of the Hakim’s triad was present, in slightly smaller percentages, in open-aqueduct LOVA. On the other hand, it was less frequent in LIAS, with gait disturbances found in 73.9% of patients, urinary incontinence in 43.5% and cognitive impairment in only 39.1%. Thus, LOVA could be recognized as a pathological entity characterized by overlapping symptoms from the other two categories of adult hydrocephalus.

### A diagnostic score

Different scales to classify and standardize hydrocephalus symptoms and radiological findings are available and validated [3, 15, 24–27, 31]. Nevertheless, a comprehensive diagnostic score including clinical and radiological features, with a clear aim on differential diagnosis of adult hydrocephalus, has never been proposed. Developing our score, we focused on the differential diagnosis of pathologies requiring different forms of surgical treatment, with the final goal of providing a practical tool to discriminate between iNPH and no iNPH cases. According to our results, a diagnostic score with such features is feasible with satisfactory accuracy (sensitivity of 95.1% and specificity of 90.6%). We purposely chose to exclude the enlarged cisterna magna and the aqueductal stenosis from our scoring system. As the first one is a constant of the LOVA cohort and the second one as part of the LIAS cohort by definition, they were used to differentiate the two included forms of obstructive hydrocephalus. Moreover, an enlarged cisterna magna is also an inconstant but not trascurable finding of the iNPH cohort. It is clear and we underline that the proposed score is a proof-of-principle of its feasibility, as it is modeled and analysed only in a small, retrospective and monocentric cohort. On the other hand, it could also represent a starting point for hydrocephalus researchers, to be improved, extended and validated in larger cohorts.

### Clinical outcome

Overall, ETV turned out to be successful in 90% of no iNPH patients, with a higher rate of symptoms improvement in LIAS as compared to LOVA (*p* = 0.02). This is in line with the findings of Ibáñez-Botella et al. and Ved et al. reporting an ETV success rate between 76 and 93% [18, 42]. These satisfactory results seem to corroborate the “obstructive” pathophysiological mechanism of no iNPH cases and support our view about the necessity of robust criteria for distinguishing iNPH from no iNPH to identify patients that may benefit from ETV.

### Strengths and limitations

The strengths of our study are that all patients included were diagnosed in a single centre, by an established hydrocephalus-focused multidisciplinary team of expert physicians. Similarly, iNPH patients were gathered from an established prospective study which ensured systematicity of the data collection and strict adhesion to diagnostic criteria according to 2005 guidelines.

There are, however, some limitations. As an unavoidable bias of the retrospective data collection, patients which lacked complete data were excluded (a significant percentage of 11.5%). A significant, but substantially unavoidable limitation of our study is the overlapping of many features of the analysed pathologies, especially between open-aqueduct LOVA and iNPH. Since the two pathologies share many tracts and there is an inevitable diagnostic “grey zone”, patients with LOVA could have been treated as iNPH patients and vice versa, despite the adhesion to diagnostic criteria established in literature [36, 38, 42]. Furthermore, our routine hydrocephalus diagnostic algorithm lacked systematical fundus examination, ICP measurement and MR-venography to investigate potential obstacles to venous outflow with impaired retrograde CSF reabsorption. The results of all of these diagnostic procedures results could have also be compared between the three cohorts, shedding more light on peculiar features of the analysed pathologies. Finally, despite iNPH incidence is significant, and it is diagnosed and treated daily on a worldwide basis, LOVA and LIAS diagnosis are not as common, even in specialized centres, leading us to analyse small sample sizes which may preclude a more thorough statistical analysis and a better generalizability of the results.

Further analysis of surgical outcome, complications and factors predicting treatment success is beyond the scope of this paper, which primarily and purposely focuses on preoperative features.

### Conclusions and implications of our study

The definitive identification of specific clinical and neuroradiological features of LOVA, LIAS and iNPH could help in defining their clinical criteria, which up to now also remains a challenge, and therefore could help to standardize the management and treatment of these patients. Despite some overlapping characteristics between the three cohorts, we suggest that typical open-aqueduct LOVA patients are on average 70 years old and significantly younger than the usual iNPH, but older than LIAS. Similarly to LIAS, they have macrocephaly and suffer from a severe degree of ventriculomegaly, sometimes with sellar bone changes or bulging of the third ventricle floor. Both LIAS and LOVA typically lack a DESH sign that is a marker of iNPH. The finding of an enlarged cisterna magna with an observed caudal cerebellar compression in LOVA caused by an hypothesized “intracisternal obstruction” appears to be a peculiar feature which can be proposed between of its diagnostic criteria. This study provides some additional evidence concerning the clinical characteristics and neuroradiological features of different forms of adult hydrocephalus. Our clinical-radiological findings should be considered in the diagnostic criteria of these misdiagnosed and heterogeneous forms of adult hydrocephalus. Moreover, according to our reports, a diagnostic score with the aim of differentiate these separate pathological entities appears to be feasible and could have a role in clinical practice, although with mandatory validation on larger cohorts. Further well-designed, prospective and multicentric studies on larger samples should be performed to improve our results.

## Supplementary Information

Below is the link to the electronic supplementary material.

Supplementary file1 (PDF 112 KB)

## Data Availability

The authors declare that the gathered data included and used for analysis outline are available in the manuscript.
