# Supplementary material for: Open-aqueduct LOVA, LIAS, iNPH: a comparative clinical-radiological study exploring the “grey zone” between different forms of chronic adulthood hydrocephalus
Source: Acta Neurochir (Wien). 2022 Apr 27;164(7):1777–88. doi: 10.1007/s00701-022-05215-9 (PMC9233635; doi:10.1007/s00701-022-05215-9)
Supplement: Supplementary file 1 — Supplementary file1 (PDF 112 KB) [file 701_2022_5215_MOESM1_ESM.pdf]

Open – aqueduct LOVA, LIAS, iNPH: a comparative clinical - radiological study exploring the "grey zone" between different forms of chronic adulthood hydrocephalus.

Giorgio Palandri[1*], Alessandro Carretta[1,2*], Emanuele La Corte[1,2], Giulia Giannini[2,3], Matteo Martinoni[1], Paolo Mantovani[1], Luca Albini-Riccioli[4], Caterina Tonon[2,5], Diego Mazzatenta[2,6], Benjamin D. Elder[7], Alfredo Conti[1,2]

* The two authors equally contributed to the manuscript.

Affiliations:

1. Department of Neurosurgery, IRCCS Istituto delle Scienze Neurologiche di Bologna, Bologna, Italy.
2. Department of Biomedical and NeuroMotor Sciences (DIBINEM), University of Bologna, Italy.
3. Neurology Unit (NEUROMET), IRCCS Istituto delle Scienze Neurologiche di Bologna, Bologna, Italy.
4. Neuroradiology Unit, IRCCS Istituto delle Scienze Neurologiche di Bologna, Bologna, Italy.
5. Functional and Molecular Neuroimaging Unit, IRCCS Istituto delle Scienze Neurologiche di Bologna, Bologna, Italy.
6. Programma Neurochirurgia Ipofisi - Pituitary Unit, IRCCS Istituto delle Scienze Neurologiche di Bologna, Bologna, Italy.
7. Department of Neurologic Surgery, Mayo Clinic, Rochester, Minnesota.

Correspondence to:

Alessandro Carretta, MD
IRCCS Istituto delle Scienze Neurologiche di Bologna, Department of Neurosurgery
Via Altura 3, 40139, Bologna, Italy.
Email: alessandro.carretta1@gmail.com

Supplementary Tables to the submission to Acta Neurochirurgica.

	Variable	Cutoff	Accuracy %	Sensitivity %	Specificity %	Youden Index (J)	OR	95% CI
LOVA VS LIAS	Age, y	59.5	39.4	88.9	52.2	0.41	1.974	1.214 - 3.211
	Cranial Circumference, cm	58.5	29.4	50	73.9	0.24	-	-
	Tentorial Angle, °	46.5	26.4	83.3	47.8	0.31	1.657	1.008 - 2.723
	Evans Index	0.365	45.8	88.9	47.8	0.37	1.974	1.214 - 3.211
	Third Ventricle Width, mm	13.5	63	88.9	60.9	0.50	2.253	1.333 - 3.810
	Callosal Angle, °	-	-	-	-	-	-	-
LOVA VS iNPH	Age, y	-	-	-	-	-	-	-
	Cranial Circumference, cm	56.5	51.8	83.3	53.1	0.36	1.320	1.100 - 1.585
	Tentorial Angle, °	46.5	13.4	83.3	45.3	0.27	-	-
	Evans Index	0.425	65.2	66.7	92.2	0.59	2.456	1.356 - 4.449
	Third Ventricle Width, mm	18.5	52.4	50	95.3	0.45	2.429	1.196 - 4.936
	Callosal Angle, °	-	-	-	-	-	-	-
LIAS VS iNPH	Age, y	-	-	-	-	-	-	-
	Cranial Circumference, cm	56.5	23.4	69.6	53.1	0.23	-	-
	Tentorial Angle, °	-	-	-	-	-	-	-
	Evans Index	0.405	18.2	39.1	84.4	0.24	-	-
	Third Ventricle Width, mm	-	-	-	-	-	-	-
	Callosal Angle, °	77.5	34.4	65.2	81.2	0.46	1.661	1.162 - 2.374

Supplementary Table 1. *Cutoff of continuous variables with percentage accuracy, sensitivity, specificity and Youden index, according to ROC curves. The two rightmost columns show the Odds Ratio with the 95% Confidence Interval of the cutoff value determined with contingency tables. AUC < 0.5 and not significant ORs were not reported.*

Variable	AUC	Cutoff	Accuracy %	Sensitivity %	Specificity %	Youden Index (J)
Age, y	0.844	72.5	68.8	79.7	80.5	0.6
Cranial circumference, cm	0.679	56.5	35.8	75.6	53.1	0.287
Evans index	0.695	0.405	39	53.7	84.4	0.381
Third ventricle width, mm	0.559	18.5	11.8	31.7	95.3	0.27
Callosal angle, °	0.44	-	-	-	-	-

Supplementary Table 2. *Cutoff between iNPH and no iNPH cohorts of continuous variables with percentage accuracy. sensitivity, specificity and Youden index, according to ROC curves. Callosal angle, with an AUC < 0.5, was excluded from the diagnostic score and its diagnostic values are not reported.*

Variable	OR	95% CI
Age, y	16.183	6.052 - 43.272
Cranial circumference, cm	3.513	1.479 - 8.348
Evans index	6.253	2.511 - 15.568
Third ventricle width, mm	9.44	2.49 - 35.792
Sellar bone distortion	17.719	2.15 - 146.052
Third ventricle floor bulging	12.39	4.127 - 37.195
DESH	190	40.282 - 896.18
Headache	20.323	2.488 - 165.977
Nausea and vomit	1.108	1.002 - 1.225
Gait disturbances	1.206	1.05 - 1.386
Urinary incontinence	5.211	1.908 - 14.236
Cognitive impairment	6.667	2.55 - 17.431

Supplementary Table 3. *ORs and 95% CI to discriminate iNPH and no iNPH cohorts of the variables included in the diagnostic score, according to contingency tables.*
